# *cis*-Aqua­chloridobis(1,10-phenanthroline-κ^2^ *N*,*N*′)cobalt(II) chloride 2.5-hydrate

**DOI:** 10.1107/S1600536809038422

**Published:** 2009-10-03

**Authors:** K. Arun Kumar, A. Dayalan, K. SethuSankar

**Affiliations:** a Department of Chemistry, Loyola College (Autonomous), Chennai-34, India; b Department of Physics, RKM Vivekananda College, Chennai-4, India

## Abstract

In the title complex, [CoCl(C_12_H_8_N_2_)_2_(H_2_O)]Cl·2.5H_2_O, the Co^II^ ion is coordinated by four N atoms of two bis-chelating 1,10-phenanthroline (phen) ligands, one water mol­ecule and a chloride ligand in a distorted octa­hedral environment. The dihedral angle between the two phen ligands is 84.21 (3)°. In the crystal structure, complex mol­ecules and chloride ions are linked into centrosymmetric four-component clusters by inter­molecular O—H⋯Cl hydrogen bonds. Of the 2.5 solvent water mol­ecules in the asymmetric unit, two were refined as disordered over two sites with fixed occupancies of ratios 0.50:0.50 and 0.60:0.40, while another was refined with half occupancy.

## Related literature

1,10-Phenanthroline is a versatile ligand capable of forming highly stable complexes with transition metal ions, see: Nobufumi (1969 ▶). Metal complexes functionalized with 1,10-phenanthrolines have been used as catalyst for the *enantio* selective hydrolysis of *N*-protected amino acid esters and in *enantio* selective reduction of acetophenone, see: Weijnen *et al.* (1992 ▶). For some examples of the applications of substituted phenanthroline compounds, see Garuti *et al.* (1989 ▶). For the crystal structures of related cobalt complexes of 1,10-phenanthroline, see: Sun & Feng (2006 ▶); Zhong *et al.* (2006 ▶). For the crystal structure of the title complex with thio­acetamide solvent rather than water, see: Zhong *et al.* (2007 ▶). For the use of metal complexes of 1,10-phenanthroline in developing new diagnostic and therapeutic agents that can recognize and cleave DNA, see: Arai *et al.* (2005 ▶); Müller *et al.* (1987 ▶). Oxovanadium complexes of dimethyl-substituted phenanthroline will induce apoptosis in human cancer cells, and may be useful for the treatment of cancer, see: Rama Krishna *et al.* (2000 ▶). Weijnen *et al.* (1992 ▶); Nobufumi (1969 ▶).
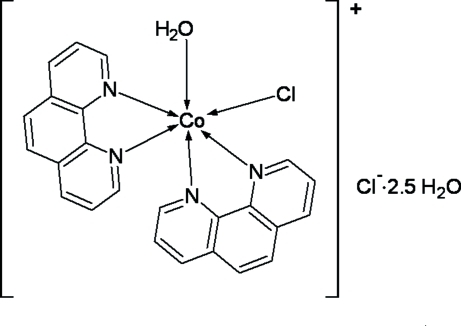


## Experimental

### 

#### Crystal data

[CoCl(C_12_H_8_N_2_)_2_(H_2_O)]Cl·2.5H_2_O
*M* *_r_* = 553.29
Triclinic, 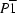

*a* = 9.6597 (3) Å
*b* = 11.4386 (3) Å
*c* = 12.9886 (4) Å
α = 64.224 (1)°
β = 86.377 (2)°
γ = 78.303 (1)°
*V* = 1265.01 (6) Å^3^
*Z* = 2
Mo *K*α radiation
μ = 0.93 mm^−1^
*T* = 293 K
0.30 × 0.30 × 0.20 mm

#### Data collection

Bruker Kappa APEXII CCD diffractometer
Absorption correction: multi-scan (*SADABS*; Sheldrick, 1996 ▶) *T*_min_ = 0.722, *T*_max_ = 0.812
34458 measured reflections
9683 independent reflections
7380 reflections with *I* > 2σ(*I*)
*R*_int_ = 0.028

#### Refinement

*R*[*F* ^2^ > 2σ(*F* ^2^)] = 0.042
*wR*(*F* ^2^) = 0.138
*S* = 1.10
9683 reflections
343 parameters
2 restraints
H atoms treated by a mixture of independent and constrained refinement
Δρ_max_ = 0.72 e Å^−3^
Δρ_min_ = −0.42 e Å^−3^

### 

Data collection: *APEX2* (Bruker, 2004 ▶); cell refinement: *SAINT* (Bruker, 2004 ▶); data reduction: *SAINT*; program(s) used to solve structure: *SIR92* (Altomare *et al*., 1993 ▶); program(s) used to refine structure: *SHELXL97* (Sheldrick, 2008 ▶); molecular graphics: *SHELXTL* (Sheldrick, 2008 ▶) and *PLATON* (Spek, 2009 ▶); software used to prepare material for publication: *SHELXL97*.

## Supplementary Material

Crystal structure: contains datablocks I, global. DOI: 10.1107/S1600536809038422/lh2895sup1.cif

Structure factors: contains datablocks I. DOI: 10.1107/S1600536809038422/lh2895Isup2.hkl

Additional supplementary materials:  crystallographic information; 3D view; checkCIF report

## Figures and Tables

**Table 1:** Hydrogen-bond geometry (Å, °)

*D*—H⋯*A*	*D*—H	H⋯*A*	*D*⋯*A*	*D*—H⋯*A*
O1—H1*A*⋯Cl2^i^	0.90 (2)	2.290 (19)	3.1530 (18)	162 (2)
O1—H1*B*⋯Cl2^ii^	0.90 (2)	2.190 (16)	3.0836 (15)	173 (3)

Symmetry codes: (i) 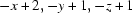; (ii) 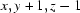.

**Fig. 1.**
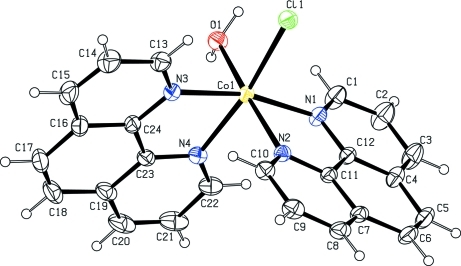
FMolecular structure of the cation of the title compound. Displacement ellipsoids are drawn at the 50% probability level.

**Fig. 2.**
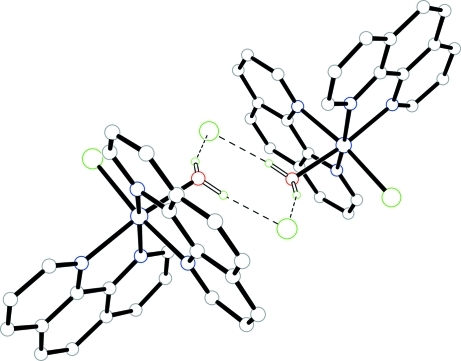
Part of the crystal structure showing O—H···Cl hydrogen bonds as dashed lines. The H atoms not involved in hydrogen bonds have been ommited. The solvent water molecules are not shown.

## supplementary crystallographic information

### Comment

1,10-phenanthroline is a versatile ligand capable of forming highly stable complexes with transition metal ions (Nobufumi, 1969). Complexes of 1,10-phenanthroline are frequently employed for catalytic reactions. For example metal complexes functionalized with 1,10-phenanthrolines have been used as catalyst for the *enantio* selective hydrolysis of N-protected amino acid esters and in *enantio* selective reduction of acetophenone (Weijnen, *et al.* 1992). The synthesis of some phenanthroline -2,9-disubstituted compounds along with their *in vitro* antimicrobial properties against gram-positive and gram – negative bacteria and fungi have been reported (Garuti *et al.*,1989). Metal complexes of 1,10-phenanthroline have been found to be attractive species for developing new diagnostic and therapeutic agents that can recognize and cleave DNA (Müller *et al.*, 1987, Arai *et al.*, 2005). Experimental evidence has been provided to prove oxovanadium complexes of dimethyl substituted phenanthroline will induce apoptosis in human cancer cells, and may be useful for the treatment of cancer (Rama Krishna, *et al.* 2000).

The molecular structure of the cation is shown in Fig. 1. The asymmetric unit contains one complex cation a chloride anion and 2.5 molecules of solvent water. The Co^II^ ion is coordinated in a distorted octahedral environment by four nitrogen atoms of two 1,10-phenanthroline ligands, a chloride ion, and a water molecule. The dihedral angle between the two phen ligands is 84.21 (3) °. In the crystal structure, complex molecules and chloride ions are linked into centrosymmetric four component clusters by intermolecular O—H···Cl hydrogen bonds. .

### Experimental

Cobalt(II) chloride hexahydrate was thoroughly grinded and exposed to microwave radiation for 30s. The dehydrated cobalt(II) chloride (0.05 mol) was dissolved in 100 ml of acetone. 1,10-phenanathroline monohydrate (0.1 mol) was dissolved in 100 ml of acetone. The solution of 1,10-phenanathroline was slowly added with constant stirring to the solution of cobalt(II) chloride and allowed to react for two hours. After completion of the reaction, a reddish orange coloured solution was formed. The stirring was stopped and the reaction mixture was allowed to settle for one hour. The reddish orange coloured product was filtered and washed with acetone and dried over a desicator. Single crystals were obtained by slow evaporation of a methanolic solution of the title complex.

### Refinement

H atoms bonded to C atoms were placed in calculated position and included in the refinement in a riding-model approximation with C-H = 0.93Å and U_iso_(H) = 1.2U_eq_(C). The H atoms bonded to the coordinated water molecule were refined with isotropic displacement parameters. Of the 2.5 solvent water molecules is the asymmetric unit two were refnied as disordered over two sites with fixed occupancies of ration 0.5:0.5 and 0.60:0.40 while another was refined as a partial occupancy of 0.50. The H atoms of the solvent water molecules were not located nor included in the refinement but were included in the molecular formula.

### Crystal data

**Table:**

[CoCl(C_12_H_8_N_2_)_2_(H_2_O)]Cl·2.5H_2_O	*Z* = 2
*M**_r_* = 553.29	*F*(000) = 576
Triclinic, *P*1	*D*_x_ = 1.463 Mg m^−^^3^
Hall symbol: -P 1	Mo *K*α radiation, λ = 0.71073 Å
*a* = 9.6597 (3) Å	Cell parameters from 7839 reflections
*b* = 11.4386 (3) Å	θ = 2.7–32.5°
*c* = 12.9886 (4) Å	µ = 0.93 mm^−^^1^
α = 64.224 (1)°	*T* = 293 K
β = 86.377 (2)°	Plate, red
γ = 78.303 (1)°	0.30 × 0.30 × 0.20 mm
*V* = 1265.01 (6) Å^3^	

### Data collection

**Table:**

Bruker Kappa APEXII CCD diffractometer	9683 independent reflections
Radiation source: fine-focus sealed tube	7380 reflections with *I* > 2σ(*I*)
graphite	*R*_int_ = 0.028
ω and φ scans	θ_max_ = 33.4°, θ_min_ = 1.7°
Absorption correction: multi-scan (*SADABS*; Sheldrick, 1996)	*h* = −14→14
*T*_min_ = 0.722, *T*_max_ = 0.812	*k* = −17→17
34458 measured reflections	*l* = −19→20

### Refinement

**Table:**

Refinement on *F*^2^	Secondary atom site location: difference Fourier map
Least-squares matrix: full	Hydrogen site location: inferred from neighbouring sites
*R*[*F*^2^ > 2σ(*F*^2^)] = 0.042	H atoms treated by a mixture of independent and constrained refinement
*wR*(*F*^2^) = 0.138	*w* = 1/[σ^2^(*F*_o_^2^) + (0.0685*P*)^2^ + 0.4443*P*] where *P* = (*F*_o_^2^ + 2*F*_c_^2^)/3
*S* = 1.10	(Δ/σ)_max_ = 0.001
9683 reflections	Δρ_max_ = 0.72 e Å^−^^3^
343 parameters	Δρ_min_ = −0.42 e Å^−^^3^
2 restraints	Extinction correction: *SHELXL97* (Sheldrick, 2008), Fc^*^=kFc[1+0.001xFc^2^λ^3^/sin(2θ)]^-1/4^
Primary atom site location: structure-invariant direct methods	Extinction coefficient: 0.0038 (12)

### Special details

**Table:**

Geometry. All e.s.d.'s (except the e.s.d. in the dihedral angle between two l.s. planes) are estimated using the full covariance matrix. The cell e.s.d.'s are taken into account individually in the estimation of e.s.d.'s in distances, angles and torsion angles; correlations between e.s.d.'s in cell parameters are only used when they are defined by crystal symmetry. An approximate (isotropic) treatment of cell e.s.d.'s is used for estimating e.s.d.'s involving l.s. planes.
Refinement. Refinement of *F*^2^ against ALL reflections. The weighted *R*-factor *wR* and goodness of fit *S* are based on *F*^2^, conventional *R*-factors *R* are based on *F*, with *F* set to zero for negative *F*^2^. The threshold expression of *F*^2^ > σ(*F*^2^) is used only for calculating *R*-factors(gt) *etc*. and is not relevant to the choice of reflections for refinement. *R*-factors based on *F*^2^ are statistically about twice as large as those based on *F*, and *R*- factors based on ALL data will be even larger.

### Fractional atomic coordinates and isotropic or equivalent isotropic displacement parameters (Å^2^)

**Table:**

	*x*	*y*	*z*	*U*_iso_*/*U*_eq_	Occ. (<1)
C1	0.6979 (2)	0.9429 (3)	0.1406 (2)	0.0498 (5)	
H1	0.7847	0.9396	0.1056	0.060*	
C2	0.6035 (3)	0.8711 (3)	0.1298 (3)	0.0634 (7)	
H2	0.6280	0.8205	0.0891	0.076*	
C3	0.4749 (3)	0.8757 (3)	0.1794 (3)	0.0593 (6)	
H3	0.4102	0.8295	0.1718	0.071*	
C4	0.4415 (2)	0.9510 (2)	0.24212 (18)	0.0413 (4)	
C5	0.3106 (2)	0.9586 (2)	0.3001 (2)	0.0485 (5)	
H5	0.2420	0.9155	0.2936	0.058*	
C6	0.2858 (2)	1.0266 (2)	0.36317 (19)	0.0444 (5)	
H6	0.2015	1.0276	0.4019	0.053*	
C7	0.38682 (18)	1.09809 (18)	0.37217 (15)	0.0355 (4)	
C8	0.3665 (2)	1.1707 (2)	0.43717 (17)	0.0426 (4)	
H8	0.2844	1.1737	0.4782	0.051*	
C9	0.4674 (2)	1.2367 (2)	0.44004 (18)	0.0436 (4)	
H9	0.4545	1.2861	0.4822	0.052*	
C10	0.5913 (2)	1.22959 (19)	0.37879 (17)	0.0377 (4)	
H10	0.6594	1.2755	0.3810	0.045*	
C11	0.51474 (17)	1.09520 (16)	0.31430 (14)	0.0300 (3)	
C12	0.54289 (17)	1.01907 (18)	0.24953 (15)	0.0319 (3)	
C13	0.9632 (2)	1.2764 (2)	0.31592 (19)	0.0430 (4)	
H13	0.9838	1.1955	0.3800	0.052*	
C14	1.0192 (3)	1.3822 (3)	0.3113 (3)	0.0567 (6)	
H14	1.0759	1.3711	0.3713	0.068*	
C15	0.9900 (3)	1.5015 (2)	0.2182 (3)	0.0565 (6)	
H15	1.0279	1.5720	0.2137	0.068*	
C16	0.9028 (2)	1.5171 (2)	0.1295 (2)	0.0449 (5)	
C17	0.8652 (3)	1.6387 (2)	0.0285 (3)	0.0590 (7)	
H17	0.9002	1.7123	0.0201	0.071*	
C18	0.7806 (3)	1.6483 (2)	−0.0541 (2)	0.0606 (7)	
H18	0.7584	1.7283	−0.1188	0.073*	
C19	0.7241 (2)	1.5380 (2)	−0.04446 (18)	0.0460 (5)	
C20	0.6344 (3)	1.5425 (3)	−0.1270 (2)	0.0594 (7)	
H20	0.6094	1.6204	−0.1933	0.071*	
C21	0.5838 (3)	1.4333 (3)	−0.1105 (2)	0.0579 (6)	
H21	0.5228	1.4362	−0.1644	0.069*	
C22	0.6249 (2)	1.3163 (2)	−0.01098 (18)	0.0444 (4)	
H22	0.5913	1.2415	−0.0009	0.053*	
C23	0.75899 (18)	1.41697 (17)	0.05320 (15)	0.0334 (3)	
C24	0.85017 (18)	1.40623 (17)	0.14098 (16)	0.0334 (3)	
O2'	0.6535 (14)	0.3986 (11)	0.6280 (11)	0.195 (6)	0.40
O2	0.3871 (11)	0.4705 (7)	0.6502 (7)	0.190 (4)	0.60
O3	0.9469 (11)	0.3167 (6)	0.6132 (6)	0.125 (3)	0.50
O3'	1.0826 (15)	0.2976 (8)	0.6422 (8)	0.180 (5)	0.50
O4	1.1914 (12)	0.4501 (10)	0.5278 (7)	0.171 (4)	0.50
N1	0.66940 (16)	1.01569 (16)	0.19855 (14)	0.0350 (3)	
N2	0.61525 (15)	1.16040 (14)	0.31806 (12)	0.0299 (3)	
N3	0.88180 (15)	1.28706 (15)	0.23231 (13)	0.0319 (3)	
N4	0.70912 (16)	1.30782 (15)	0.06895 (13)	0.0329 (3)	
O1	0.95884 (15)	1.10031 (14)	0.11464 (12)	0.0382 (3)	
Cl1	0.92546 (5)	0.95686 (5)	0.38472 (4)	0.03919 (11)	
Cl2	0.88362 (6)	0.18004 (6)	0.86264 (5)	0.04968 (13)	
Co1	0.79827 (2)	1.13428 (2)	0.222016 (18)	0.02766 (7)	
H1A	1.006 (3)	1.0167 (13)	0.138 (2)	0.062 (8)*	
H1B	0.930 (3)	1.129 (3)	0.0417 (11)	0.058 (8)*	

### Atomic displacement parameters (Å^2^)

**Table:**

	*U*^11^	*U*^22^	*U*^33^	*U*^12^	*U*^13^	*U*^23^
C1	0.0438 (11)	0.0636 (14)	0.0639 (14)	−0.0216 (10)	0.0158 (10)	−0.0447 (12)
C2	0.0605 (14)	0.0834 (18)	0.0834 (19)	−0.0336 (13)	0.0197 (13)	−0.0640 (17)
C3	0.0521 (13)	0.0754 (17)	0.0757 (17)	−0.0336 (12)	0.0103 (12)	−0.0476 (15)
C4	0.0346 (9)	0.0504 (11)	0.0434 (10)	−0.0180 (8)	0.0024 (7)	−0.0203 (9)
C5	0.0328 (9)	0.0598 (13)	0.0524 (12)	−0.0216 (9)	0.0032 (8)	−0.0187 (10)
C6	0.0258 (8)	0.0540 (11)	0.0452 (11)	−0.0110 (7)	0.0057 (7)	−0.0134 (9)
C7	0.0270 (7)	0.0379 (8)	0.0308 (8)	−0.0038 (6)	0.0030 (6)	−0.0062 (7)
C8	0.0337 (9)	0.0462 (10)	0.0366 (9)	−0.0005 (7)	0.0083 (7)	−0.0114 (8)
C9	0.0455 (10)	0.0461 (10)	0.0385 (10)	−0.0022 (8)	0.0072 (8)	−0.0212 (8)
C10	0.0383 (9)	0.0393 (9)	0.0371 (9)	−0.0065 (7)	0.0037 (7)	−0.0188 (7)
C11	0.0253 (7)	0.0319 (7)	0.0263 (7)	−0.0047 (5)	0.0001 (5)	−0.0069 (6)
C12	0.0270 (7)	0.0362 (8)	0.0313 (8)	−0.0094 (6)	0.0004 (6)	−0.0119 (6)
C13	0.0411 (10)	0.0462 (10)	0.0478 (11)	−0.0080 (8)	−0.0065 (8)	−0.0251 (9)
C14	0.0518 (12)	0.0613 (14)	0.0757 (17)	−0.0144 (11)	−0.0087 (11)	−0.0440 (13)
C15	0.0506 (12)	0.0496 (12)	0.0876 (18)	−0.0215 (10)	0.0092 (12)	−0.0424 (13)
C16	0.0410 (10)	0.0345 (9)	0.0619 (13)	−0.0133 (7)	0.0157 (9)	−0.0226 (9)
C17	0.0592 (14)	0.0306 (9)	0.0793 (18)	−0.0159 (9)	0.0251 (13)	−0.0165 (10)
C18	0.0700 (16)	0.0306 (9)	0.0575 (14)	−0.0056 (9)	0.0199 (12)	−0.0016 (9)
C19	0.0458 (10)	0.0358 (9)	0.0371 (10)	0.0030 (8)	0.0101 (8)	−0.0040 (7)
C20	0.0609 (14)	0.0527 (13)	0.0353 (10)	0.0138 (11)	−0.0021 (10)	−0.0023 (9)
C21	0.0543 (13)	0.0698 (16)	0.0361 (10)	0.0118 (12)	−0.0160 (9)	−0.0184 (11)
C22	0.0410 (10)	0.0521 (11)	0.0378 (10)	0.0014 (8)	−0.0075 (8)	−0.0206 (9)
C23	0.0313 (8)	0.0306 (7)	0.0312 (8)	−0.0017 (6)	0.0070 (6)	−0.0094 (6)
C24	0.0306 (7)	0.0305 (7)	0.0386 (9)	−0.0075 (6)	0.0086 (6)	−0.0149 (7)
O2'	0.207 (12)	0.110 (7)	0.169 (10)	−0.002 (7)	0.033 (9)	0.015 (7)
O2	0.282 (10)	0.100 (5)	0.180 (7)	−0.020 (5)	−0.061 (7)	−0.051 (5)
O3	0.238 (9)	0.059 (3)	0.062 (3)	−0.024 (5)	0.029 (5)	−0.017 (2)
O3'	0.349 (16)	0.074 (5)	0.123 (7)	−0.049 (8)	0.064 (9)	−0.053 (5)
O4	0.242 (10)	0.192 (9)	0.128 (6)	−0.078 (8)	0.062 (7)	−0.107 (7)
N1	0.0306 (7)	0.0418 (8)	0.0396 (8)	−0.0130 (6)	0.0071 (6)	−0.0221 (7)
N2	0.0283 (6)	0.0313 (6)	0.0281 (6)	−0.0055 (5)	0.0021 (5)	−0.0114 (5)
N3	0.0306 (7)	0.0323 (7)	0.0332 (7)	−0.0076 (5)	0.0009 (5)	−0.0138 (6)
N4	0.0329 (7)	0.0350 (7)	0.0289 (7)	−0.0032 (5)	0.0017 (5)	−0.0137 (6)
O1	0.0384 (7)	0.0389 (7)	0.0338 (7)	−0.0044 (5)	0.0073 (5)	−0.0147 (5)
Cl1	0.0372 (2)	0.0397 (2)	0.0311 (2)	−0.00573 (16)	−0.00006 (16)	−0.00724 (16)
Cl2	0.0557 (3)	0.0504 (3)	0.0480 (3)	−0.0067 (2)	0.0007 (2)	−0.0273 (2)
Co1	0.02642 (11)	0.02914 (11)	0.02756 (12)	−0.00822 (8)	0.00243 (8)	−0.01140 (8)

### Geometric parameters (Å, °)

**Table:**

C1—N1	1.328 (3)	C14—H14	0.9300
C1—C2	1.394 (3)	C15—C16	1.397 (4)
C1—H1	0.9300	C15—H15	0.9300
C2—C3	1.365 (4)	C16—C24	1.406 (3)
C2—H2	0.9300	C16—C17	1.433 (3)
C3—C4	1.405 (3)	C17—C18	1.343 (4)
C3—H3	0.9300	C17—H17	0.9300
C4—C12	1.401 (2)	C18—C19	1.428 (4)
C4—C5	1.437 (3)	C18—H18	0.9300
C5—C6	1.338 (3)	C19—C20	1.397 (4)
C5—H5	0.9300	C19—C23	1.406 (3)
C6—C7	1.434 (3)	C20—C21	1.359 (4)
C6—H6	0.9300	C20—H20	0.9300
C7—C8	1.401 (3)	C21—C22	1.401 (3)
C7—C11	1.407 (2)	C21—H21	0.9300
C8—C9	1.360 (3)	C22—N4	1.318 (3)
C8—H8	0.9300	C22—H22	0.9300
C9—C10	1.403 (3)	C23—N4	1.357 (2)
C9—H9	0.9300	C23—C24	1.432 (3)
C10—N2	1.322 (2)	C24—N3	1.353 (2)
C10—H10	0.9300	N1—Co1	2.1389 (15)
C11—N2	1.354 (2)	N2—Co1	2.1453 (14)
C11—C12	1.432 (3)	N3—Co1	2.1241 (15)
C12—N1	1.354 (2)	N4—Co1	2.1738 (15)
C13—N3	1.329 (2)	O1—Co1	2.1108 (13)
C13—C14	1.398 (3)	O1—H1A	0.896 (10)
C13—H13	0.9300	O1—H1B	0.898 (10)
C14—C15	1.363 (4)	Cl1—Co1	2.3835 (5)
			
N1—C1—C2	122.9 (2)	C17—C18—C19	121.3 (2)
N1—C1—H1	118.5	C17—C18—H18	119.4
C2—C1—H1	118.5	C19—C18—H18	119.4
C3—C2—C1	119.5 (2)	C20—C19—C23	117.1 (2)
C3—C2—H2	120.2	C20—C19—C18	123.8 (2)
C1—C2—H2	120.2	C23—C19—C18	119.1 (2)
C2—C3—C4	119.1 (2)	C21—C20—C19	120.1 (2)
C2—C3—H3	120.5	C21—C20—H20	120.0
C4—C3—H3	120.5	C19—C20—H20	120.0
C12—C4—C3	117.69 (18)	C20—C21—C22	119.1 (2)
C12—C4—C5	119.22 (19)	C20—C21—H21	120.4
C3—C4—C5	123.07 (19)	C22—C21—H21	120.4
C6—C5—C4	121.13 (18)	N4—C22—C21	122.7 (2)
C6—C5—H5	119.4	N4—C22—H22	118.6
C4—C5—H5	119.4	C21—C22—H22	118.6
C5—C6—C7	121.14 (18)	N4—C23—C19	122.64 (19)
C5—C6—H6	119.4	N4—C23—C24	117.64 (15)
C7—C6—H6	119.4	C19—C23—C24	119.72 (18)
C8—C7—C11	117.27 (17)	N3—C24—C16	122.97 (19)
C8—C7—C6	123.58 (17)	N3—C24—C23	117.24 (15)
C11—C7—C6	119.15 (18)	C16—C24—C23	119.78 (18)
C9—C8—C7	119.68 (17)	C1—N1—C12	117.90 (16)
C9—C8—H8	120.2	C1—N1—Co1	128.30 (13)
C7—C8—H8	120.2	C12—N1—Co1	113.80 (12)
C8—C9—C10	119.29 (19)	C10—N2—C11	118.18 (15)
C8—C9—H9	120.4	C10—N2—Co1	128.29 (13)
C10—C9—H9	120.4	C11—N2—Co1	113.53 (11)
N2—C10—C9	122.77 (19)	C13—N3—C24	117.96 (16)
N2—C10—H10	118.6	C13—N3—Co1	127.18 (13)
C9—C10—H10	118.6	C24—N3—Co1	114.83 (12)
N2—C11—C7	122.79 (17)	C22—N4—C23	118.33 (17)
N2—C11—C12	117.62 (14)	C22—N4—Co1	128.70 (14)
C7—C11—C12	119.59 (16)	C23—N4—Co1	112.74 (12)
N1—C12—C4	122.85 (17)	Co1—O1—H1A	116.3 (19)
N1—C12—C11	117.43 (15)	Co1—O1—H1B	114.6 (18)
C4—C12—C11	119.72 (16)	H1A—O1—H1B	107 (3)
N3—C13—C14	122.6 (2)	O1—Co1—N3	93.44 (6)
N3—C13—H13	118.7	O1—Co1—N1	94.48 (6)
C14—C13—H13	118.7	N3—Co1—N1	166.38 (6)
C15—C14—C13	119.6 (2)	O1—Co1—N2	171.52 (6)
C15—C14—H14	120.2	N3—Co1—N2	93.79 (6)
C13—C14—H14	120.2	N1—Co1—N2	77.58 (6)
C14—C15—C16	119.52 (19)	O1—Co1—N4	85.36 (5)
C14—C15—H15	120.2	N3—Co1—N4	77.19 (6)
C16—C15—H15	120.2	N1—Co1—N4	92.42 (6)
C15—C16—C24	117.4 (2)	N2—Co1—N4	91.91 (5)
C15—C16—C17	123.7 (2)	O1—Co1—Cl1	90.18 (4)
C24—C16—C17	118.9 (2)	N3—Co1—Cl1	96.39 (4)
C18—C17—C16	121.3 (2)	N1—Co1—Cl1	94.66 (5)
C18—C17—H17	119.4	N2—Co1—Cl1	93.43 (4)
C16—C17—H17	119.4	N4—Co1—Cl1	171.92 (4)
			
N1—C1—C2—C3	0.7 (5)	C9—C10—N2—Co1	−178.68 (14)
C1—C2—C3—C4	−1.1 (5)	C7—C11—N2—C10	−0.1 (2)
C2—C3—C4—C12	0.6 (4)	C12—C11—N2—C10	−179.13 (16)
C2—C3—C4—C5	−178.1 (3)	C7—C11—N2—Co1	179.45 (13)
C12—C4—C5—C6	−1.8 (3)	C12—C11—N2—Co1	0.39 (18)
C3—C4—C5—C6	176.9 (2)	C14—C13—N3—C24	−1.5 (3)
C4—C5—C6—C7	2.1 (3)	C14—C13—N3—Co1	176.37 (17)
C5—C6—C7—C8	−179.9 (2)	C16—C24—N3—C13	1.9 (3)
C5—C6—C7—C11	−0.6 (3)	C23—C24—N3—C13	−178.24 (17)
C11—C7—C8—C9	1.4 (3)	C16—C24—N3—Co1	−176.25 (14)
C6—C7—C8—C9	−179.36 (19)	C23—C24—N3—Co1	3.6 (2)
C7—C8—C9—C10	−0.8 (3)	C21—C22—N4—C23	−0.7 (3)
C8—C9—C10—N2	−0.3 (3)	C21—C22—N4—Co1	−174.82 (17)
C8—C7—C11—N2	−1.0 (3)	C19—C23—N4—C22	0.1 (3)
C6—C7—C11—N2	179.73 (16)	C24—C23—N4—C22	179.86 (17)
C8—C7—C11—C12	178.05 (16)	C19—C23—N4—Co1	175.15 (14)
C6—C7—C11—C12	−1.2 (3)	C24—C23—N4—Co1	−5.10 (19)
C3—C4—C12—N1	0.5 (3)	C13—N3—Co1—O1	−98.21 (17)
C5—C4—C12—N1	179.22 (19)	C24—N3—Co1—O1	79.76 (12)
C3—C4—C12—C11	−178.9 (2)	C13—N3—Co1—N1	136.3 (2)
C5—C4—C12—C11	−0.1 (3)	C24—N3—Co1—N1	−45.7 (3)
N2—C11—C12—N1	1.3 (2)	C13—N3—Co1—N2	86.24 (17)
C7—C11—C12—N1	−177.80 (16)	C24—N3—Co1—N2	−95.79 (12)
N2—C11—C12—C4	−179.35 (17)	C13—N3—Co1—N4	177.34 (17)
C7—C11—C12—C4	1.6 (3)	C24—N3—Co1—N4	−4.69 (12)
N3—C13—C14—C15	0.1 (4)	C13—N3—Co1—Cl1	−7.64 (17)
C13—C14—C15—C16	1.0 (4)	C24—N3—Co1—Cl1	170.32 (12)
C14—C15—C16—C24	−0.7 (3)	C1—N1—Co1—O1	4.1 (2)
C14—C15—C16—C17	179.6 (2)	C12—N1—Co1—O1	−175.11 (13)
C15—C16—C17—C18	−179.8 (2)	C1—N1—Co1—N3	129.5 (3)
C24—C16—C17—C18	0.4 (3)	C12—N1—Co1—N3	−49.7 (3)
C16—C17—C18—C19	0.3 (4)	C1—N1—Co1—N2	−179.0 (2)
C17—C18—C19—C20	179.3 (2)	C12—N1—Co1—N2	1.85 (13)
C17—C18—C19—C23	−0.5 (3)	C1—N1—Co1—N4	89.6 (2)
C23—C19—C20—C21	0.6 (3)	C12—N1—Co1—N4	−89.58 (13)
C18—C19—C20—C21	−179.1 (2)	C1—N1—Co1—Cl1	−86.5 (2)
C19—C20—C21—C22	−1.1 (4)	C12—N1—Co1—Cl1	94.34 (13)
C20—C21—C22—N4	1.2 (4)	C10—N2—Co1—O1	−160.7 (3)
C20—C19—C23—N4	−0.1 (3)	C11—N2—Co1—O1	19.8 (4)
C18—C19—C23—N4	179.69 (18)	C10—N2—Co1—N3	−12.39 (16)
C20—C19—C23—C24	−179.81 (18)	C11—N2—Co1—N3	168.16 (12)
C18—C19—C23—C24	−0.1 (3)	C10—N2—Co1—N1	178.27 (17)
C15—C16—C24—N3	−0.8 (3)	C11—N2—Co1—N1	−1.18 (11)
C17—C16—C24—N3	178.91 (19)	C10—N2—Co1—N4	−89.67 (16)
C15—C16—C24—C23	179.33 (18)	C11—N2—Co1—N4	90.87 (12)
C17—C16—C24—C23	−0.9 (3)	C10—N2—Co1—Cl1	84.26 (15)
N4—C23—C24—N3	1.1 (2)	C11—N2—Co1—Cl1	−95.20 (11)
C19—C23—C24—N3	−179.10 (16)	C22—N4—Co1—O1	85.00 (17)
N4—C23—C24—C16	−179.01 (16)	C23—N4—Co1—O1	−89.41 (12)
C19—C23—C24—C16	0.7 (3)	C22—N4—Co1—N3	179.59 (18)
C2—C1—N1—C12	0.3 (4)	C23—N4—Co1—N3	5.19 (11)
C2—C1—N1—Co1	−178.9 (2)	C22—N4—Co1—N1	−9.31 (17)
C4—C12—N1—C1	−0.9 (3)	C23—N4—Co1—N1	176.29 (12)
C11—C12—N1—C1	178.44 (19)	C22—N4—Co1—N2	−86.96 (17)
C4—C12—N1—Co1	178.38 (15)	C23—N4—Co1—N2	98.64 (12)
C11—C12—N1—Co1	−2.3 (2)	C22—N4—Co1—Cl1	141.7 (3)
C9—C10—N2—C11	0.7 (3)	C23—N4—Co1—Cl1	−32.7 (4)

### Hydrogen-bond geometry (Å, °)

**Table:**

*D*—H···*A*	*D*—H	H···*A*	*D*···*A*	*D*—H···*A*
O1—H1A···Cl2^i^	0.90 (2)	2.29 (2)	3.1530 (18)	162 (2)
O1—H1B···Cl2^ii^	0.90 (2)	2.19 (2)	3.0836 (15)	173 (3)

Symmetry codes: (i) −*x*+2, −*y*+1, −*z*+1; (ii) *x*, *y*+1, *z*−1.
